# Insertion torque recordings for the diagnosis of contact between orthodontic mini-implants and dental roots: protocol for a systematic review

**DOI:** 10.1186/s13643-015-0014-6

**Published:** 2015-04-02

**Authors:** Reint Meursinge Reynders, Luisa Ladu, Laura Ronchi, Nicola Di Girolamo, Jan de Lange, Nia Roberts, Annette Plüddemann

**Affiliations:** 1 Department of Oral and Maxillofacial Surgery, Academic Medical Center, University of Amsterdam, Meibergdreef 9, 1105 AZ Amsterdam, The Netherlands; 2 Private practice of orthodontics, Via Matteo Bandello 15, 20123 Milan, Italy; 3 Department of Veterinary Sciences, University of Bologna, Via Tolara di Sopra 50, Ozzano dell’Emilia (BO), 40064 Italy; 4 Department of Oral and Maxillofacial Surgery, Academic Medical Center and Academisch Centrum Tandheelkunde Amsterdam (ACTA), University of Amsterdam, Meibergdreef 9, Amsterdam, AZ 1105 The Netherlands; 5 Bodleian Health Care libraries, University of Oxford, Cairns Library Level 3, John Radcliffe Hospital, Oxford, OX3 9DU UK; 6 Department of Primary Care Health Sciences, Centre for Evidence-Based Medicine, University of Oxford, New Radcliffe House, 2nd floor, Jericho, Oxford, OX2 6NW UK

**Keywords:** Diagnostic test accuracy, Implant, Screw, Root contact, Root proximity, Insertion torque, Orthodontics, Systematic review

## Abstract

**Background:**

Hitting a dental root during the insertion of orthodontic mini-implants (OMIs) is a common adverse effect of this intervention. This condition can permanently damage these structures and can cause implant instability. Increased torque levels (index test) recorded during the insertion of OMIs may provide a more accurate and immediate diagnosis of implant-root contact (target condition) than radiographic imaging (reference standard). An accurate index test could reduce or eliminate X-ray exposure. These issues, the common use of OMIs, the high prevalence of the target condition, and because most OMIs are placed between roots warrant a systematic review. We will assess 1) the diagnostic accuracy and the adverse effects of the index test, 2) whether OMIs with root contact have higher insertion torque values than those without, and 3) whether intermediate torque values have clinical diagnostic utility.

**Methods:**

The Preferred Reporting Items for Systematic review and Meta-Analysis Protocols (PRISMA-P) 2015 statement was used as a the guideline for reporting this protocol. Inserting implants deliberately into dental roots of human participants would not be approved by ethical review boards and adverse effects of interventions are generally underreported. We will therefore apply broad spectrum eligibility criteria, which will include clinical, animal and cadaver models. Not including these models could slow down knowledge translation.

Both randomized and non-randomized research studies will be included. Comparisons of interest and subgroups are pre-specified. We will conduct searches in MEDLINE and more than 40 other electronic databases. We will search the grey literature and reference lists and hand-search ten journals. All methodological procedures will be conducted by three reviewers. Study selection, data extraction and analyses, and protocols for contacting authors and resolving conflicts between reviewers are described. Designed specific risk of bias tools will be tailored to the research question. Different research models will be analysed separately. Parameters for exploring statistical heterogeneity and conducting meta-analyses are pre-specified. The quality of evidence for outcomes will be assessed through the Grading of Recommendations Assessment, Development and Evaluation (GRADE) approach.

**Discussion:**

The findings of this systematic review will be useful for patients, clinicians, researchers, guideline developers, policymakers, and surgical companies.

**Electronic supplementary material:**

The online version of this article (doi:10.1186/s13643-015-0014-6) contains supplementary material, which is available to authorized users.

## Background

Orthodontic mini-implants (OMIs) are used to provide anchorage during orthodontic tooth movement. Contact between these devices and dental roots during implant insertion is a common problem, because inter-radicular spaces are narrow [1-6]. Such contacts have been associated with damage of these structures and increased implant failure rates [7-9]. Because most OMIs are placed between dental roots and the prevalence of implant-root contact is high, an accurate test for the diagnosis of this target condition is indicated [3,5,10].

### Target condition being diagnosed

To control the reciprocal forces of tooth movement, orthodontists need some form of anchorage. It is usually obtained by connecting these forces to groups of teeth in the same or the opposing jaw or through the use of intra-or extra-oral removable appliances. However, these treatment mechanics may still cause anchorage loss, have a limited area of application, or depend on patient collaboration [11]. OMIs are not conditioned by most of these limitations, but they need to be inserted surgically.

A 2014 survey of orthodontists in the USA by the *Journal of Clinical Orthodontics* identified that 90% of OMIs are inserted at inter-radicular sites [10]. Contact between OMIs and dental roots is one of the risks of this intervention and is the target condition of this systematic review. This condition can cause extensive damage to the roots [7,12]. Studies in maxillofacial surgery have reported similar problems with intermaxillary fixation screws [13-15]. The quality of the healing of the root injury as a result of the target condition varies and damage involving the dental pulp is less likely to repair completely [7,12,16]. Additional root damage can occur during orthodontic treatment, because implants are not stable and they can migrate towards dental roots [17-19]. This issue is particularly important, because contact between implants and the periodontal ligament was identified in 65.2% of consecutively inserted OMIs [20]. Close contact between OMIs and dental roots has also been associated with increased failure rates of these devices [3,9,21-23]. A recent systematic review identified three times higher failure rates in OMIs with root contact compared with those placed away from adjacent roots [8].

**Figure 1:**
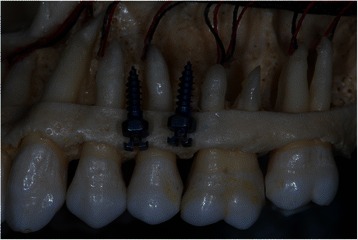
Inter-radicular distances in the maxillary arch and 1.5 mm (diameter) orthodontic mini-implants. Quattro implants PSM Medical Solutions; Tuttlingen, Germany.

The diameters of the most commonly used OMIs vary between 1.2 and 2 mm [8]. Only little space for error is possible, because inter-radicular distances of at least 3 mm have been recommended for safe placement of OMIs. Such dimensions are only available in limited areas of the dental arches (Figure 1) [4,6]. Specific surgical and radiographic positioning techniques have been developed to avoid root contact during implant insertion [24-27]. These methods are accurate, but they require additional radiographs and complex and expensive surgical guides [3,28,29].

Various studies have assessed the prevalence of the target condition. Cho *et al*. [2] scored the target condition in 21.3% of the implant insertions for inexperienced operators and in 13.5% for experienced operators. Kim *et al*. [3] scored 30% for implant-root contact, and Motoyoshi *et al*. [5] identified a prevalence of 17.1% for screws that were inserted with the self-drilling technique and 20.5% for those that were pre-drilled. This high prevalence of the target condition strengthens the importance of accurately diagnosing implant-root contact.

### Reference standard

**Figure 2:**
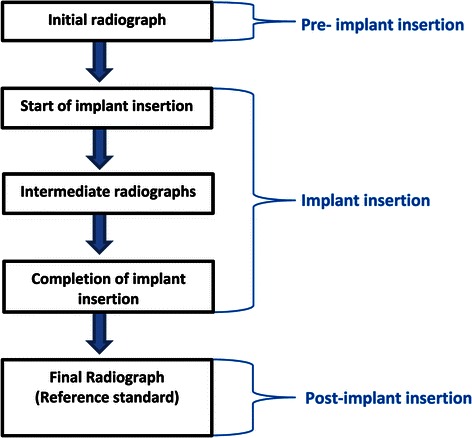
Current diagnostic pathway for assessing implant-root contact.

In current practice, X-rays are used to measure inter-radicular distances prior to implant placement (Figure 2). Additional radiographs are taken to diagnose implant-root contact during implant insertion and at the completion of this procedure. The latter assessment is the reference standard of the current diagnostic pathway. No other reference standards are currently used to diagnose the target condition.

Studies that have used either two-dimensional or three-dimensional images are both eligible for our clinical questions, but the latter type is more accurate, because it also provides information on the third dimension [30,31]. Although X-rays are often considered the ‘gold’ standard of discriminative power, they can still produce false positive and false negative outcomes [32,33]. However, the main disadvantage of this method is the exposure to radiation. This is particularly a problem for three-dimensional radiographs, which produce higher radiation exposure than two-dimensional imaging [34].

### The index test

The recording of torque values during the insertion of OMIs does not have these shortcomings and is the index test for our clinical questions. The American Society for Testing and Materials (ASTM International) defines insertion torque as ‘the amount of torque required to overcome the frictional force between the screw and the material used for testing while driving the screw into the material’ [35]. This index test was chosen after having addressed the following preliminary questions [36]:

1. Is the prevalence of the target condition sufficiently high to order the diagnostic test?
2. Is there an effective treatment for the target condition?
3. Could the test introduce a change in the management strategies that is beneficial to the patients?
4. Are patients undergoing the new test expected to be better off than those who do not from a health perspective?
5. Are the costs and cost-effectiveness analyses of the new test expected to justify implementation?

All five questions were answered with a ‘Yes’.

Advantages of the index test could include the following: (1) increasing the certainty of diagnosing root contact. In various animal models, increased insertion torque values have been associated with the target condition [16,37,38]; (2) reducing or eliminating the use of the current reference standard and therefore the exposure to X-rays; and (3) contributing to the decision-making process, because the continuous recordings of torque during the entire insertion process could inform the clinician at which specific time point the OMI touches the root. A sudden steep increase in torque values could indicate root contact [37].

Potential disadvantages associated with the index test could include increased costs, a steep learning curve, a longer diagnostic pathway, pain and discomfort, and unforeseen adverse effects. However, most of these disadvantages are not applicable, because the index test does not: (1) prolong the procedure; (2) require additional learning (3) or introduce pain and discomfort, because torque values are measured during the current standard insertion process with sensors that are built into the screwdrivers. The index test will probably also reduce costs in the long run, because taking less or no radiographs will shorten the duration and lower the costs of the current diagnostic pathway. Furthermore, the purchasing price of the diagnostic instrument is relatively low (±€1,000), and this device can also be used for other diagnostic purposes, for example, measuring implant stability [9,39].

The test under review could be a candidate for a role as a ‘replacement’, a ‘triage’, or as an ‘add-on’ test [40,41]. It could replace the reference standard if the index test is, for example, more accurate, cheaper, faster, and causing less adverse effects (for example, less exposure to radiation) than the reference standard. Both intermediate and final radiographs can then be avoided. A role as a triage test (minimize false negatives) to minimize the use of the more expensive and invasive reference standard could be indicated, for example, in young cancer patients that have undergone radiotherapy. The index test could also be used as an add-on test to improve accuracy.

The importance for conducting the index test was explained in the previous paragraphs. Scoping searches of the literature did not identify any review that assessed the clinical utility of the index test. Systematic reviews that critically appraise the literature and address specific research questions on this topic are therefore indicated.

### Objectives

#### Primary objectives

For the primary objectives of this systematic review, the diagnostic accuracy question is formulated according to the participants, index test, reference standard, target condition (PIRT) mnemonic [42,43]:

- In OMIs (participants or problem), what is the accuracy of the level of insertion torque values (index test) compared to radiography (reference standard) to distinguish those with and without implant-root contact (target condition).

#### Secondary objectives

For the secondary objectives, we formulated the following questions:

- ‘Do OMIs with root contact have higher insertion torque values than those without this target condition?’ ‘We will also assess whether intermediate recordings of insertion torque values have clinical utility for the diagnosis of the target condition’.

## Methods/design

The guideline for conducting systematic reviews of diagnostic accuracy of the Diagnostic Test Accuracy Working Group of the Cochrane Collaboration and the Cochrane Handbook for Systematic reviews of Interventions will be adopted to address our research questions [44,45]. The Preferred Reporting Items for Systematic review and Meta-Analysis Protocols (PRISMA-P) 2015 statement is used as a the guideline for reporting this protocol [46,47]. Differences in methodology between research questions are explained for each of these items. Changes in the methods during the conduct of this systematic review from those outlined in this protocol will be fully reported.

### Eligibility criteria

Our eligibility criteria will be adapted to the particular character of our research questions, because we do not expect to find clinical studies in which implants are deliberately inserted into dental roots. Because ethical review boards do not approve such procedures, we will also include *in vivo* animal studies and cadaver models. Including these experimental models is important because: 1) animal and cadaver studies might provide additional information on the usefulness of conducting the index test; 2) they could provide information on how to design future research studies on our clinical question; 3) considering outcomes from animal studies avoids wasting valuable research information, financial resources, and duplication [48,49]. The importance of these issues were further stressed by Iain Chalmers, one of the founders of the Cochrane Collaboration, in a recent international symposium on systematic reviews in laboratory animal science [50]; and 4) not considering these studies would risk that knowledge creation on this topic would remain at a standstill. These issues are further strengthened in the context of the high prevalence of the target condition, the risk of biologic damage of the interventional procedure, the instability of implants with the target condition, and the underreporting of adverse effects of interventions [7-9,20,51,52]. Actually, one could reason that it would be unethical not to include experimental studies when only limited numbers of clinical studies will be identified.

This systematic review is not registered in the PROSPERO database, because its inclusion criteria cover only studies on human participants [53]. To avoid inappropriate exclusion of relevant articles, we will aim for more broad-scope inclusion criteria that are sufficiently specific and still cover all research objectives [54].

### Study designs

- Our preferred research design will be studies that randomize participants to either creating or not creating implant-root contact. We do not expect to find randomized studies because (1) this research design is rare in diagnostic accuracy studies and (2) of the ethical reasons outlined in the previous section.
- Non-randomized studies (NRS), that is, cohort (prospective and retrospective) and case-control study designs, that used the index test alone or in combination with the reference standard and compared outcomes on participants with or without the target condition are eligible [55,56]. The decision to include NRS in systematic reviews is based on the justifications presented by the Non-Randomized Studies Methods Group of the Cochrane Collaboration and the Oxford 2011 Levels of Evidence Working Group [56-58]. These rationales include the following: (1) high-quality NRS could produce less biased outcomes compared to low-quality randomized controlled trials (RCTs); (2) RCTs are not always needed; (3) ethical reasons could make RCTs unfeasible; (4) NRS could reveal the deficiencies of the current literature and show the need of additional research; (5) findings of these NRS could help in designing such studies; (6) NRS could reveal unexpected, rare, or long-term harmful effects of interventions [56,58,59]; and (7) large magnitude of treatment effects, the presence of a dose response gradient, or plausible residual confounding could upgrade the validity of NRS [60,61].
- Cadaver and animal studies will also be included. The choice for including such study models was explained in the previous section.
- Non-primary studies such as ‘editorials’, ‘view point publications’, ‘case reports’, ‘reviews’, and studies that used computer simulated models, for example, finite element analyses, are excluded.
- Eligibility will not be based on ‘design labels’ only, because studies can be poorly indexed or labels can be used inconsistently by authors [56,62].
- Outcomes of different study designs are assessed separately.

### Participants

- Adults and adolescents of 12 years and older of either sex, in any ethnicity, setting, or socio-economic group, no medical or dental history, in need of stationary anchorage during orthodontic treatment with fixed appliances are our preferred participants to include.
- For the animal studies, only monkeys and dogs are eligible, because these animal models are mostly used in orthodontic research and their dentitions closely resemble those of humans. For the cadaver studies, both human and animal models will be eligible. For each research model, outcomes will be assessed separately.
- Participants that had undergone a previous surgical intervention or the index test in the same area, for example, placement of an additional OMI or re-implantation of OMIs in the same site, are excluded, because such procedures could affect outcomes.

### Interventions

- The target condition of interest is contact between OMIs and dental roots, which is currently diagnosed with the reference standard. This target condition refers to single or multiple implant-root contacts with or without root penetration [5,37,38].
- The recordings of insertion torque values during the insertion of OMIs in inter-radicular areas of the maxillary and mandibular alveolar bone will be the index test under investigation.

### Outcomes

- Insertion torque values for OMIs with and without root contact will be eligible for both primary and secondary research questions. These outcomes are collected as reported in the original studies [47]. Only torque units that are convertible to Newton centimeter (Ncm) are eligible.
- Recordings with either mechanical or digital torque drivers are eligible for the primary research question. We decided to include both types of index tests, because the Diagnostic Test Accuracy Working Group of the Cochrane Collaboration recommends avoiding index test criteria that are too narrow [54]. Subsequent analyses could then assess differences between these subgroups.
- For the secondary research objective, we will also assess whether sudden steep increases in torque values during the implant insertion process were identified. Such an outcome could indicate implant-root contact. Only recordings with digital torque sensors are eligible, because mechanical sensors cannot record continuous torque values and can therefore not assess how torque measures change during implant insertion.
- Eligibility will not be influenced by the outcome of the test, that is, negative outcomes are considered as important as positive outcomes.
- Both two- and three-dimensional radiographs are eligible as the reference test for the primary and secondary research questions.
- For the secondary research questions, histology will also be eligible as the reference standard.
- Because the degree of error between reference standards might differ, their outcomes will be assessed separately in subgroups.

### Timing

- Torque recordings at various time points are eligible. Variations in these domains between studies will be assessed in subgroup analyses.
- The time points of recording the reference standards have to be identical as those recorded for the index test.

### Setting

We will not apply restrictions by type of setting. Participants treated in either teaching or non-teaching settings are therefore eligible.

### Language

No language restrictions will be applied and pertinent non-English articles will be translated.

### Information sources

#### Electronic searches

Eligible studies will be searched in the period from 1 January 1997, the year of the introduction of orthodontic mini implants in orthodontics, onwards [63]. The following protocol will be applied to find eligible studies:

- General and subject-specific electronic databases will be consulted from PubMed (MEDLINE), Google Scholar Beta, Embase (Ovid), Science Direct, and Cochrane Central Register of Controlled Trials (CENTRAL) [62,64,65].
- Additional studies will be searched through the ‘Related Articles’ feature in PubMed.
- Databases for diagnostic studies: TRIP Database, NHS Evidence, and SUMSearch2 will also be searched.
- The following citation indexes will be searched: Science Citation Index, Scopus, and Web of Science [62,64].
- The following national and regional databases will also be searched: African Index Medicus, African Journals online (AJOL), Australasian Medical Index, Index Medicus for the Eastern Mediterranean Region, IndMED, KoreaMed, LILACS, Index Medicus for the South-East Asia Region (IMSEAR), and Western Pacific Region Index Medicus (WPRIM) [62,64].

### Searching other resources

#### Grey literature

Eligible reports are also searched in the grey literature, for example, research registers, conference proceedings, and university dissertations [64-66]. The following grey databases will be searched:

- General databases: Google Scholar Beta, Open Grey, The Health Management Information Consortium (HMIC), and The National Technical Information Service (NTIS) [62].
- Dissertations and databases of theses: ProQuest Dissertations & Databases, Index to Theses in Great Britain and Ireland, and DissOnline [62].
- Conference abstracts or proceedings: Conference Proceedings Citation Index (Web of Science), BIOSIS Citation Index, Meeting Abstracts, and ISI Proceedings [62].
- Review databases: Meta-analyses van Diagnostisch Onderzoek (MEDION), Database of Abstracts of Reviews of Effects (DARE), Health Technology Assessment database (HTA), and Turning Research into Practice (TRIP) [62].
- MEDLINE, Embase, and TRIP are searched for guidelines. In addition, evidence-based guidelines of the following organizations are consulted: Australian National Health and Medical Research Council, Canadian Medical Association, National Guideline Clearinghouse, National Library of Guidelines, New Zealand Guidelines Group, and NICE Clinical Guidelines [62].
- Citations alerts in PubMed and Ovid [62].

#### Handsearching

- American Journal of Orthodontics and Dentofacial Orthopedics, Angle Orthodontist, Australian Journal of Orthodontics, European Journal of Orthodontics, International Journal of Adult Orthodontics and Orthognathic surgery, Journal of Clinical Orthodontics, Journal of Orthodontics, Journal of the World Federation of Orthodontics, Orthodontics, Orthodontics & Craniofacial Research, Progress in Orthodontics, and Seminars in Orthodontics.

#### Reference lists

- Each selected paper, all review articles, and guidelines will be screened manually for references of relevant articles that possibly are not identified in the searches of the electronic databases [62,64].

#### Correspondence

- Subject specialists, authors of the selected articles, and researchers and manufacturers involved in our topic of interest will be contacted to identify unpublished or ongoing studies [62,67].

#### Search strategy

- A librarian (NR) specialized in computerized searches of healthcare publications will assist with the development of the search strategy.
- A detailed protocol for developing this search strategy is presented in Additional file 1 [62,64,68-71].
- Pertinent search terms are summarized in Table 1.

**Table 1:** **Search terms for the index test and the target condition**

Item	Search terms
Index test	Torque OR insertion torque OR torquing OR torqueing OR torque sensor OR torque device OR torquing device OR torqueing device OR torque screwdriver OR torque driver
Target condition (1)	Root OR root contact OR root vicinity OR dental root OR root damage OR tooth OR teeth OR tooth contact OR tooth vicinity
Target condition (2)	Implant OR mini implant OR micro implant OR microimplant OR screw OR mini screw OR miniscrew OR micro screw OR microscrew OR temporary anchorage device
- Search strategies will be pilot tested for each database and subsequently fine-tuned [62]. Examples are presented for the search strategy of MEDLINE and Google Scholar in Table 2 [64,70].

**Table 2:** **Search strategy for the MEDLINE and Google Scholar Beta databases** ^a^

Database	Search strategy
PubMed (MEDLINE)	(torque OR insertion torque OR torquing OR torqueing OR torque sensor* OR torque device* OR torquing device* OR torqueing device* OR torque screwdriver* OR torque driver*) AND (root* OR root contact* OR root vicinity OR dental root* OR root damage OR tooth OR teeth OR tooth contact* OR tooth vicinity) AND (implant* OR mini implant* OR micro implant* OR microimplant* OR screw* OR mini screw* OR miniscrew* OR micro screw* OR microscrew* OR temporary anchorage device*)
Google Scholar Beta^b^	(orthodontics OR orthodontic OR orthodontist OR orthodontists) (torque OR torquing) (root OR roots OR tooth OR teeth) (implant OR implants OR “mini implant” OR screw OR screws OR ‘mini screw’ OR miniscrew OR ‘temporary anchorage device’)

^a^The appropriate characters for the truncation and exploration of search terms are adapted for each individual database. To avoid errors during the transfer of search terms, all search strategies are copied and pasted from the original without re-typing [64]. ^b^This shortened search strategy was chosen, because Google Scholar limits its search strings to under 256 characters [70].
- We will list the search strategies for each database in a table together with the total number of records retrieved and the search dates.

### Study records

#### Data management

To reduce the risk of inter-examiner disagreement, we will adopt the protocol presented in the Cochrane Handbook for Systematic Reviews of Interventions [72]. A sample of articles will be first pilot tested to refine and clarify the eligibility criteria and to apply them consistently [72]. These procedures will be conducted by three review authors (RMR, LL, and LR).

#### Selection process

- Three topic experts (RMR, LL, and LR) will independently select the studies.
- Titles and abstracts will be screened for eligibility, and the full texts of potentially relevant articles will be retrieved and subsequently reviewed. To avoid inappropriate exclusion, ambiguous articles will also be read.
- Unpublished research studies, for example, those extracted from the grey literature, which contain sufficient data to permit peer-reviewing will be reviewed independently by the topic experts. When data of unpublished studied are insufficient for adequate peer-reviewing, authors of these papers will be contacted. Our protocol for contacting authors is described in Additional file 2 [73,74]. When no additional data will be retrieved, outcomes of such reports are only used in the discussion to put effect estimates of eligible studies in perspective.
- Authors, suspect of multiple publications of the same research study will also be contacted (Additional file 2). Suspicion of multiple publications is based on the following characteristics: (1) studies with a retrospective design with similar methodology; (2) same authors in similar research studies; and (3) publication of similar findings in different journals within a short time span.
- In the case of disagreement on the eligibility of an article, the review authors will discuss the selection procedures, reread the paper, and if necessary, contact its authors [67] (Additional file 2).
- A PRISMA flow diagram will illustrate the selection procedures and excluded articles will be presented in a table together with the rationale for their exclusion [67,75].

#### Data collection process

Data collection forms (see ‘Data items’ subsection) will be first pilot tested on their validity and subsequently fine-tuned. These procedures are also used as calibration exercises and are conducted independently by three experienced systematic reviewers (RMR, LL, and LR). Disagreements will be resolved through discussions. An arbitrator (NDG) will be consulted to adjudicate remaining disagreements.

#### Data items

- The Standards for the Reporting of Diagnostic accuracy studies (STARD) checklist was consulted for the development of data extraction forms [76,77]. Data collection forms of previous systematic reviews on OMIs were also checked for pertinent items [73,78,79]. All data collection forms are tailored to our specific research questions.
- Pilot tested collection forms are presented in Additional file 3 and address both our primary and secondary research questions. These forms are prepared in a sequence that facilitates the application of the Quality Assessment of Diagnostic Accuracy Studies (QUADAS)-2 tool [80]. This instrument assesses risk of bias and concerns of applicability of outcomes of individual studies in diagnostic accuracy studies. If modifications of these forms will be necessary during the review process, we will report these changes and will explain them.
- Flow diagrams of participants will be created for each individual study.
- Data from multiple publications of the same study will be first extracted from each article. These data will be subsequently analysed for potential overlap.
- All data extraction procedures will be conducted independently by three topic experts (RMR, LL, and LR).
- Disagreement between reviewers on extracted data will be resolved through discussions and rereading. If necessary, authors will be contacted for clarification (Additional file 2).
- An arbitrator (NDG) will be consulted to resolve remaining disagreements.
- Persisting disagreements between reviewers will be reported.
- We will apply the protocol presented by the Cochrane Handbook for Systematic Reviews of Interventions for dealing with missing data, for example, missing outcomes, summary data, individuals, or study-level characteristics [81]. We will first apply our protocol for contacting authors (Additional file 2). When authors do not reply or are unable to provide us with this information, we will assess whether data were missing at random or not. The rationale and consequences for all assumptions and methods for dealing with missing data will be addressed in the discussion [81]. A statistician will be consulted for selecting an appropriate statistical model. All imputation techniques are avoided whenever possible. Sensitivity analyses will be conducted to assess how reasonable changes in assumptions affect results [81].

#### Outcomes and prioritization

To establish a threshold for test positivity, we conducted scoping searches of the literature. We identified one clinical and one animal study during these initial searches [5,16]. Insertion torque values of OMIs with root contact in self-drilling groups increased respectively 22.5% in human participants and 113% in adult beagles compared with implants without this target condition [5,16,]. Based on these findings, we will define for our primary outcomes a hypothetical maximum insertion torque increase of 25% or more as a positive result of the index test and values inferior to this threshold as a negative outcome. Maximum insertion torque values for OMIs with and without root contact will therefore be recorded for the primary outcomes.

Differences in the type of target condition, for example, with or without root penetration, and different time points and insertion depths for measuring these outcomes will be subdivided and assessed separately.

The difference between maximum insertion torque recordings will be calculated for the secondary outcomes. We will also record whether sudden steep increases in torque values were identified during the implant insertion process. Outcomes measures will be recorded in the original format as defined by the authors of the selected studies. These measures will be transformed to the effect estimate of this systematic review, that is, Ncm, after the completion of all data extraction procedures [72].

#### Risk of bias individual studies

**Figure 3:**
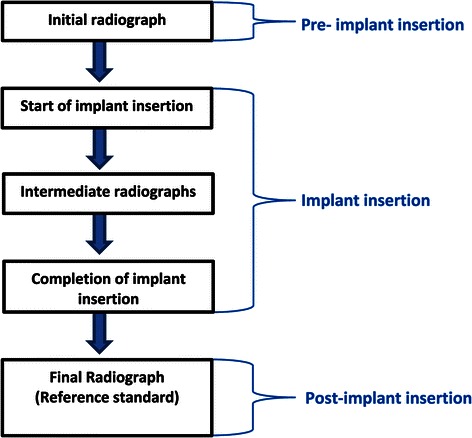
Tailoring the QUADAS-2 tool to the systematic review [82].

For the primary research question, we will adopt the QUADAS-2 tool [80]. The content and the rating guidelines of the QUADAS-2 tool are tailored to our review question according to the protocol depicted in a flow diagram (Figure 3) [82]. If indicated, certain signaling questions were added and others were omitted. To standardize the application of assessment criteria, clear definitions for each criterion were established prior to conducting the review [83]. The revised tool was subsequently pilot tested on a small number of eligible studies. This process was conducted by the three topic experts (RMR, LL, and LR). A methodologist, AP, was consulted for assistance and to serve as a referee. The tailored and pilot tested QUADAS-2 tool is presented in Additional file 4. This tool will be subsequently applied to all eligible studies. A table will be used to display the QUADAS-2 assessments (Additional file 5) [80,82]. QUADAS-2 scores are not used to generate a summary ‘quality score’, because such scores can be problematic [82,84,85].

**Figure 4:**
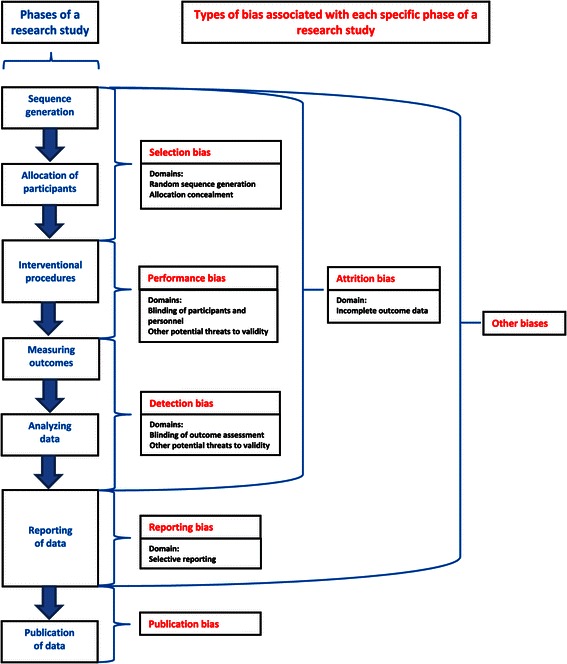
The six types of bias of the Cochrane ‘Risk of bias tool’ and publication bias [86].

**Figure 5:**
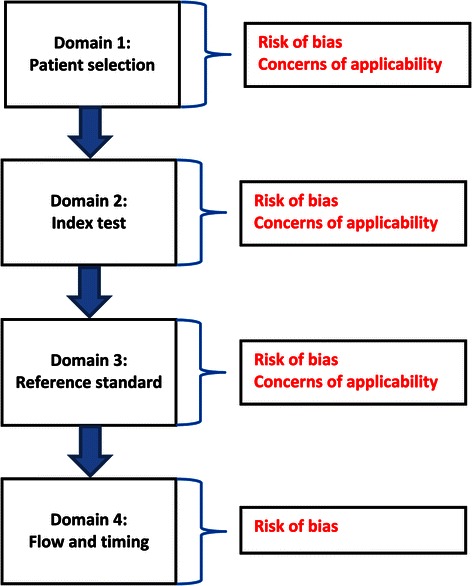
QUADAS-2 judgments on bias and applicability [82].

For the secondary research questions, we will use the ‘Risk of bias tool’ of the Cochrane collaboration, because this instrument has been designed specifically for interventional questions [86]. Both the Cochrane and the QUADAS-2 tools use domain-based evaluations of systematic error, but their biases are defined differently and cover different parts of the research phases. The differences between these tools are illustrated in Figures 4 and 5 [82,86]. Figure 4 also shows how the various phases of the research study are covered by specific types of bias. Risk of bias will be scored as ‘High’, ‘Low’, or ‘Unclear’. The Cochrane Handbook for Systematic Reviews of Interventions assigns this latter judgement if: 1) ‘insufficient detail is reported of what happened in the study’; 2) ‘what happened in the study is known, but the risk of bias is unknown’; 3) ‘an entry is not relevant to the study at hand’. The assessments of risk of bias will be presented in a ‘Risk of bias summary figure’ [86].

The scoring for both the QUADAS-2 and the Cochrane Risk of Bias tools will be conducted by three review authors (RMR, LL, and LR). Disagreements will be resolved through discussions. In the case of persisting disagreements, the methodologist, AP, will serve as an arbitrator. If necessary, authors of selected studies will also be contacted for clarification [87]. Risk of bias scores will be used to assess their potential influence on the overall outcomes of the review and on the confidence in the cumulative estimate, for example, for the Grading of Recommendations Assessment, Development and Evaluation (GRADE) judgments [47]. Risk of bias scores will also be consulted during the decision-making whether to undertake a meta-analysis or not (see data synthesis).

### Data synthesis

#### Qualitative synthesis

We will present and explain the characteristics and outcomes of the eligible studies in a narrative synthesis. This qualitative summary will be conducted whether or not a quantitative data synthesis will be considered appropriate. Characteristics of included studies will be presented first and outcomes are subsequently listed according to the order of our research questions [47]. This narrative summary will be completed with a series of tables: (1) characteristics of participants; (2) characteristics of selection procedures; (3) characteristics of the target condition, index test, and reference standard; (4) tabular presentation of the QUADAS-2 results; (5) tabular presentation of the Cochrane risk of bias assessment; (6) outcomes of the diagnostic accuracy tests; (7) insertion torque values with or without implant-root contact; (8) adverse effects of interventions; and (9) information obtained from contacted authors.

#### Data analysis

For the primary outcomes of each selected study, we will score our binary outcomes in 2 × 2 contingency tables, which present the outcomes of the test results and those of the reference standard. The specificity, sensitivity, the positive and negative predictive values of the index test, and the positive and negative likelihood ratios will be calculated for each of these studies. We will also calculate the number needed to diagnose and misdiagnose. The diagnostic odds ratio will be calculated when outcomes of various studies are synthesized in a meta-analysis. Besides the 25% threshold for test positivity, we will also assess thresholds at respectively 15%, 35%, 45%, and 55% and will present them in receiver operating characteristic (ROC) curves [88]. *Q* values will not be used in this systematic review, because they frequently give a wrong impression of accuracy [88].

For the secondary research questions, the mean insertion torque values with their standard deviation for OMIs with and without root contact will be presented for each selected study. The mean difference between these recordings will be calculated. These values will be reported along with the 95% confidence intervals. These effect measures will be presented in a forest plot. Clinical and experimental studies are presented in separate figures. Statistical tests will be carried out with Review Manager version 5.3 [89]. All intervention groups of multi-arm studies will be listed in the table ‘Characteristics of included studies’.

Unit of analysis issues could arise according to the level at which randomization occurs or in studies with repeated recordings of insertion torque values [90]. These issues will be analysed for each specific study design, and our primary analysis will be per randomized individual [47,90].

A meta-analysis is conducted in the case of the following: 1) low risk of bias in the selected studies, 2) consistent outcomes across the various studies, 3) low publication bias, 4) a high number of eligible studies, and 5) low heterogeneity [86,90,91]. Our protocol for conducting a meta-analysis for the primary and secondary research questions is presented in Additional file 6 [88-99].

#### Subgroup analysis and investigation of heterogeneity

Sources of heterogeneity that could affect outcomes can be categorized as study design, participants, target condition, index test, reference standard, implant, location of insertion, surgery, and setting related factors [78,79,88]. In this systematic review, we plan to investigate the following potential sources of heterogeneity:

- Study design: randomized versus non-randomized studies
- Study participants: participants between the ages of 12 and 18 versus participants of 18 years and older
- The type of implant
- The type of implant insertion: pre-drilling versus self-drilling insertion techniques
- The type of index test: digital versus mechanical torque sensors
- The type of reference standard: two-dimensional versus three-dimensional radiographs
- The type of target condition: implant-root contact with root penetration versus without root penetration
- The time point for measuring outcomes

Heterogeneity between research models, that is, clinical, animal, and cadaver studies, will not be assessed, because these models are analysed separately.

The following protocol will be applied for dealing with heterogeneity [90,94,100-102]:

1. Tables are created for each of the *a priori* defined sources of heterogeneity. Data are extracted and compiled in these tables, and the correctness of these procedures is subsequently double-checked. For both research questions, we will assess the same sources of heterogeneity.
2. If necessary, authors of selected research studies are contacted for clarification of specific issues.
3. The data extraction tables and the forest plots are subsequently inspected to assess whether it makes sense to apply statistics and further explore heterogeneity.

Subgroup analyses and meta-regression will be used to investigate statistical heterogeneity [90]. A statistician will be consulted for both statistics. We will report whether a subgroup analysis was planned *a priori* or was undertaken *post hoc*. Subgroup analyses will only be performed if sufficient data are reported for such assessments [90]. Meta-regressions will be conducted to investigate whether effect sizes are associated with specific characteristics of the study, for example, randomized sequence generation, and blinding of personnel. These analyses are only undertaken when a minimum of ten studies can be modelled for specific characteristics [90]. The limitations of both subgroup analyses and meta-regressions are considered when interpreting these statistics [103]. Models for investigating statistical heterogeneity are presented in Additional file 6 [88-99].

#### Sensitivity analysis

Sensitivity analyses will assess whether decisions made during the undertaking of the systematic review did affect its findings. Decision nodes that are pre-specified for our sensitivity analysis include the following: (1) the inclusion of grey literature or unpublished studies, (2) mechanical versus digital torque sensors, (3) the threshold for test positivity of 25% or more, (4) the inclusion of unblinded studies, and (5) the definition of the reference standard [88].

Additional sensitivity analyses will be conducted when specific issues suitable for such an analysis will arise during the review process [88]. All *post hoc* decisions to undertake such analyses will be reported.

#### Meta-biases

To assess the presence of reporting bias, we will assess whether protocols of trials are available and whether they were published prior to recruiting participants [47]. The Clinical Trial Register at the International Clinical Trials Registry Platform of the World Health Organization will be searched to identify such studies published after 1 July 2005 [104]. We will evaluate whether outcomes that were planned in the protocols were actually reported on in the published studies. Selective reporting of outcomes in all the eligible studies is also assessed as well as bias as a result of the outcomes of smaller studies. We will measure whether the random effects model presents more beneficial outcomes for the smaller studies than the fixed effect estimate [47]. Outcomes with or without data obtained from contacted authors will also be compared. The effect of including grey literature or unpublished studies on outcomes will be assessed in sensitivity analyses. Funnel plots will be conducted to further explore reporting bias [65,105]. Asymmetry in the funnel plots will only be assessed when ten or more eligible studies are identified, because with fewer articles the power of this statistics is too low [65]. All procedures to assess the meta-biases will be conducted by three review authors (RMR, LL, and LR).

#### Confidence in cumulative estimate/assessment of the quality of evidence (GRADE)

Judgments about the quality of the evidence for the primary research question are rated according to the GRADE protocol (Table 3) [106-109]. The GRADEpro software will be used for the completion of this protocol, and the QUADAS-2 scores are integrated into this assessment [110].

**Table 3:** **The four levels of evidence** [106]

Level of evidence	Description
High quality	We are very confident that the true effect lies close to that of the estimate of the effect
Moderate quality	We are moderately confident in the effect estimate: the true effect is likely to be close to the estimate of the effect, but there is a possibility that it is substantially different
Low quality	Our confidence in the effect estimate is limited: the true effect may be substantially different from the estimate of the effect
Very low quality	We have very little confidence in the effect estimate: the true effect is likely to be substantially different from the estimate of effect

For the secondary question, we assessed whether OMIs with root contact have higher insertion torque values than those without this target condition. Because this question does not specifically addresses a health problem, it does not qualify for an assessment using the GRADE approach.

## Discussion

The strengths of this systematic review are as follows: 1) it will be conducted by experienced reviewers, who have produced several systematic reviews and commentaries on this research topic [73,78,79,111,112]; and 2) it will be based on extensive literature searches, which will aim for high sensitivity and will accept low precision [64].

A weakness of this systematic review could be the eligibility of non-randomized studies and the inclusion of animal and cadaver models. However, we presented numerous reasons for placing this issue in a different perspective. These models could provide important knowledge for clinical applications and future research studies. Not considering these studies could waste valuable information and money, could slow down knowledge creation on this topic, and could lead to unnecessary duplication of research [48-50,56-58].

As outlined above, it could also be viewed as unethical not to include these research models. This issue was further strengthened in the context of the 1) high prevalence of the target disorder and the associated biologic damage and implant instability [7-9,20]; 2) ethical limitations of designing clinical studies on this research topic; 3) overwhelming evidence that data on adverse effects of interventions are poorly reported in the literature [51,52]; 4) the wide application of this interventional procedure in both orthodontics and maxillofacial surgery [10,13-15]; and 5) the usefulness of the research information for a wide group of stakeholders, that is, clinicians, researchers, patients in need of orthodontic implants or intermaxillary fixation screws, guideline developers, policymakers, and companies that produce implants and surgical instruments.

Proposals for possible future research studies will be presented, taking in consideration, the inadequacies of the selected studies, patient-important outcomes, the setting, costs, the learning curve for the operator, and other barriers to the implementation of this health technology.

## Additional files

Additional file 1: **Protocol for the search strategy.** A detailed protocol for developing this search strategy.
Additional file 2: **Protocol for contacting authors.** Protocol and sample email for contacting authors.
Additional file 3: **Data collection forms.** Pilot tested collection forms used for this protocol.
Additional file 4: **QUADAS-2 quality assessment tool tailored to the clinical question [**80**,**^82^**].** The tailored and pilot tested QUADAS-2 tool.
Additional file 5: **Tabular presentation for QUADAS-2 results of the selected studies [**80**].** Exemplary table for the presentation of the QUADAS-2 scores.
Additional file 6: **Protocols for conducting meta-analyses and assessing statistical heterogeneity.** Protocol for conducting a meta-analysis for the primary and secondary research questions and models for investigating statistical heterogeneity.

## Abbreviations

OMIs: orthodontic mini implants
RCTs: randomized controlled trials
