# Supplementary material for: Insertion torque recordings for the diagnosis of contact between orthodontic mini-implants and dental roots: protocol for a systematic review
Source: Syst Rev. 2015 Apr 2;4:39. doi: 10.1186/s13643-015-0014-6 (PMC4407834; doi:10.1186/s13643-015-0014-6)
Supplement: Additional file 1: — Protocol for the search strategy. A detailed protocol for developing this search strategy. [file 13643_2015_14_MOESM1_ESM.docx]

**Additional file 1.**

**Protocol for the search strategy**

- We will aim for a broad-spectrum search strategy, because this systematic review addresses different review questions and because diagnostic accuracy studies are often poorly reported and lack appropriate subject headings [62]. The search strategies for the various databases will therefore be highly sensitive, i.e., low risk of missing relevant studies, but with low precision, i.e. high number of irrelevant papers [62]. We will not apply “methodological or other search filters” to avoid the exclusion of pertinent papers [62,68]. Diagnostic search filters are currently not reliable when searching studies for systematic reviews [69].
- Search strategies for articles on diagnostic test accuracy typically use two sets of search terms: terms related to the index test(s) and those related to the target condition(s) [62]. However, the target condition “implant-root contact” of our clinical question has not been defined precisely in the orthodontic literature. We therefore subdivided the target condition in 2 sets of search terms: 1) those that refer to root contact and 2) those that refer to implants.
- The following protocol was used to find pertinent subject headings and key words : 1) conduct preliminary searches to retrieve some relevant articles or related reviews to avoid relying exclusively on controlled vocabulary terms alone [69]; 2) use the specific search tools of individual databases, e.g The “Related Articles” option in PubMed, to find additional relevant articles; 3) identify key concepts, e.g., the index test, the target condition, and the description of the patients in these papers; 4) create search terms through the identification of synonyms, related terms, acronyms, abbreviations, variant spellings, and subject headings for these key concepts. Database thesauri are also used for this purpose; 5) create subject headings that database indexers have assigned to these articles. The identified search terms are summarized in table 1.
- The Boolean operator ‘AND’ will be used for the key concepts and the search terms of each of these concepts will be combined by the Boolean operator ‘OR’. To avoid inappropriate exclusion of relevant studies we will not use The “NOT” operator.
- We will pilot test these strategies for each database and subsequently fine-tune them [62]. Examples are presented for the search strategy of MEDLINE and Google Scholar in Table 2 [64,70].
- Search strategies for each database will be listed in a table, which presents the number of records retrieved with the specific start and end dates of the searches. This table will facilitate future updating of this systematic review and improve the transparency of the search methods [67].
- Fraudulent studies, other retracted publications, errata and comments will be searched in MEDLINE [62,71].
