# Supplementary material for: Insertion torque recordings for the diagnosis of contact between orthodontic mini-implants and dental roots: protocol for a systematic review
Source: Syst Rev. 2015 Apr 2;4:39. doi: 10.1186/s13643-015-0014-6 (PMC4407834; doi:10.1186/s13643-015-0014-6)
Supplement: Additional file 2: — Protocol for contacting authors. Protocol and sample email for contacting authors. [file 13643_2015_14_MOESM2_ESM.docx]

**Additional file 2.**

**Protocol for contacting authors**

During the study selection and data extraction and analysis procedures, authors of primary research studies will be contacted to obtain additional information on eligibility issues or unclear or missing data. For this purpose the following protocol will be applied: (1) an email will be sent to the pertinent authors to ask their willingness to provide such information; (2) a second email will be sent in case of no reply; (3) if necessary, a third email will be sent from a different email address. This decision was made to avoid the risk of an email address being classified as “spam mail” by the internet provider [73]; (4) if emails are still not answered, at least one of the co-authors of the pertinent research study will also be contacted; (5) questions will be presented as a combination of open-and closed-ended questions. An example of our initial email is presented under here.

The information obtained from the contacted investigators and the consequences of this information on e.g. outcomes or risk of bias assessments will be summarized in a table. Because the validity of making author contact is currently unknown, all information obtained through the “contacting authors” procedures will be first analyzed and reported separately from the data extracted from the original articles [74].

**Email example for contacting authors**

| Dear Professor ………….,  We are currently conducting a systematic review* on orthodontic mini implants and would like to obtain some additional information on your article:    “ …………………………………………………………….”  In the near future we would like to ask you some simple questions on this publication. Answering these questions will probably not require more than 2 minutes of your time.  In this email we are only interested to find out whether you are willing to provide us with this additional information or not.  We therefore ask you the following question:  **Are you in the near future willing to respond to some simple questions pertinent to the indicated article ?**  - If you are willing to respond to our questions please reply to this email, a "YES" is enough.    - If you are not willing to respond to our questions please respond this email with a “NO”. In case, you do not reply, we will send you a reminder email 14 days after the current email.  -In case you respond with a “NO” or do not reply to this email and to the reminder, we will not further contact you, but will contact at least one of the other co-authors of your research study.  Thank you very much for your cooperation.  Sincerely,  Reint Meursinge Reynders and Nicola Di Girolamo  ----------------------------------------------------- *This systematic review originated as an assignment for the Evidence Based Health Care program at the University of Oxford, UK. The aforementioned individuals are subjectively responsible for the information requested. |
| --- |
