# Supplementary material for: Insertion torque recordings for the diagnosis of contact between orthodontic mini-implants and dental roots: protocol for a systematic review
Source: Syst Rev. 2015 Apr 2;4:39. doi: 10.1186/s13643-015-0014-6 (PMC4407834; doi:10.1186/s13643-015-0014-6)
Supplement: Additional file 3: — Data collection forms. Pilot tested collection forms used for this protocol. [file 13643_2015_14_MOESM3_ESM.docx]

**Additional file 3**

**Data collection forms**

**Data collection form 1. Source and eligibility [56,72]**

| **Entry** | **Description and character of the information** |
| --- | --- |
| Name of reviewer | Report the name of the reviewer. |
| Authors, title, journal of article | List authors, title, and journal. |
| Source of article | Describe how the article was retrieved, e.g. Medline, grey literature, hand searching of review articles etc. |
| Language of the article | Describe in which language the article was published. |
| Trial register | Describe if trial was registered, under what number and in which register. |
| Eligible/not eligible | Confirm eligibility.  If not eligible, explain the reason for exclusion. |
| Purpose | Copy the objectives of the paper according to the authors. |

**Data collection form 2. The target condition and primary outcomes [72]***

| **Entry** | **Description and character of the information** |
| --- | --- |
| Target condition | Present the definition of the target condition according to the authors, e.g., no root contact, single or multiple root contact without root penetration (glancing), root contact with root penetration. |
| Unit of measurement  Primary outcome | Describe the unit of measurement of measuring torque of the primary outcome, e.g., Newton centimeter (Ncm). |
| Time point of outcome assessment  Primary outcome | Describe the time point of the primary outcome assessment, e.g. maximum insertion torque during the entire insertion process or measured during the last turn of the OMI. |

*Locate where information on each item can be found, e.g., Page 12 column 3.

**Data collection form 3. Results [72]***

| **Entry** | **Description and character of the information** |
| --- | --- |
| Study design and selection procedures | e.g. consecutively treated randomized patients. |
| Setting | e.g. private practice, laboratory setting, university clinic. |
| Country and location | Indicate the country and the city where the study was conducted. |
| Characteristics of the population and number of participants and implants | e.g. Self-drilling group: n = 50 (70 implants) 20 males and 20 females mean age 22.3 ± 7.9 years  Pre-drilling group: n= 50 (70 implants) 20 males and 20 females mean age 23.6 ± 8.1 years  If indicated summarize the division of experimental groups, e.g. number of patients or implants per trial arm or subgroup. |
| Implant type | Indicate the type (tapered or cylindrical etc.), the diameter, and length of the implant and the company. |
| Location of insertion | e.g. between maxillary first molar and second bicuspid. |
| Insertion technique | Self-drilling and pre-drilling. |
| Index test | Present the type and the company of the index test and indicate whether the torque sensor is mechanical or digital. |
| Torque measurement | e.g., Maximum insertion torque (MIT) during the terminal rotation of the screw. |
| Pre-specified torque threshold | Present the cut-off point for implant-root contact, e.g. 50% increase in MIT. |
| Reference standard | Present the type and company of the reference standard. |
| Definition of the target condition | No root contact, root contact without penetration (glancing), root contact with penetration. |
| Prevalence of implant-root contact | Present the prevalence of implant-root contact with the percentages for the different target conditions, e.g., with or without root penetration. |
| Accuracy | Present the accuracy of the index test |
| Maximum insertion torque values with or without implant-root contact | Self-drilling 1 point contact: 8.0 ± 3.0 Ncm  Self-drilling multiple contacts: 9.5 ± 2.3 Ncm  Pre-drilling 1 point contact: 5.4 ± 1.3 Ncm  Pre-drilling multiple contacts: 7.4 ± 2.1 Ncm |
| Adverse effects | Present ratios of adverse effects of interventions, e.g. implant fracture ratios (fractured implants/total number of inserted implants). Score adverse effects according to ‘Data collection form 4’. |

*Locate where information on each item can be found, e.g., Page 12 column 3.

**Data collection form 4. Adverse effects [72] ***

| **Entry** | **Description and character of the information** |
| --- | --- |
| Biologic damage | Define identified biologic damage and how it is scored [79]. |
| Implant fracture | Describe for each subgroup the number of implants that fractured during their insertion and removal. Present the fracture ratio (the number of fractured implants/total number of implants). |
| Inflammation | Define identified inflammation and how it is scored [79].re the type of biologic damage ain and discomforttries |
| Pain and discomfort | Define identified pain and discomfort and how they are scored [79]. |
| Summary critique | Summarize the strength and weaknesses of adverse effects in the research study. |

*Locate where information on each item can be found, e.g., Page 12 column 3.

**Data collection form 5. Miscellaneous [72] ***

| **Entry** | **Description and character of the information** |
| --- | --- |
| Funding source | Describe the funding source and indicate if it is an implant company. |
| Key conclusions of study authors | Describe the main conclusions by the authors of the research study. |
| Important comments by the study authors | Describe important comments by the authors of the research study. |
| References to other relevant studies | List references of other relevant studies that should be retrieved. |
| Key conclusions of the review authors | Describe the main conclusions by the review authors. |
| Major strength according to review authors | Describe the major points of strength of the research study according to the review authors. |
| Major weaknesses according to review authors | Describe the major points of weakness of the research study according to the review authors. |
| Contacting authors | Indicate whether authors should be contacted and indicated what additional information is necessary. |
| Discuss with statistician | Describe points that need to be discussed with a statistician. |
| Points of interest | Describe points of interest, e.g. the description of a new type of index test. |
| Future studies | Describe ideas for future studies suggested by the authors or reviewers. |
| Other comments by review authors | Any additional comment that has not been covered. |

*Locate where information on each item can be found, e.g., Page 12 column 3.

**QUADAS-2 Collection form 1. Domain 1. Patient selection “risk of bias” [56,72,82]***

| **Entry** | **Description and character of the information** |
| --- | --- |
| Date of the study | Describe when the study was started and completed. |
| Study design | Describe the type of study design, e.g. randomized controlled trial, case-control study, cohort, splitmouth etc. ? |
| Consecutively treated | Describe whether participants were consecutively treated or not. |
| Sequence generation | 1) Random sequence generation ? Describe the type of randomization, e.g. computerized.  2) Non-random sequence generation ? |
| Allocation concealment | Concealment of allocation. Describe blinding of patients, surgeon, test operators, and personnel. |
| Case-control design | Describe whether patients were enrolled according to a case-control design. |
| Power calculation | Describe how power calculation was conducted. |
| Inappropriate exclusions | Describe whether single patients or specific groups of patients were inappropriate excluded during any phase of the patient selection process. |
| Approved by ethical board | Describe if the research study was approved by an ethical board and describe the components of this ethical board. |

*Locate where information on each item can be found, e.g., Page 12 column 3.

**QUADAS-2 Collection form 2. Domain 1: Patient selection “Concerns of applicability”**

**[56,72,82]***

| **Entry** | **Description and character of the information** |
| --- | --- |
| Number | Present the total number of patients and the number of patients per subgroup. |
| Ethnicity | Which ethnic group(s) ? If applicable, present the number of patients per subgroup. |
| Socio-economic status | Describe the socio-economic status of the patient(s)? If applicable, present the number of patients per subgroup. |
| Sex | Present the divisions of the sexes in the research study. If applicable, present the number of patients per subgroup. |
| Age | List age in years and months with standard deviations and/or ranges. If applicable, present the number of patients per subgroup. |
| Additional patient characteristics | Describe additional characteristics, e.g. university or high school students, dental students etc. If applicable, present the number of patients per subgroup. |
| Country | Describe in which country(ies) the research study was conducted. If applicable, present the number of patients per subgroup. |
| Medical and dental health condition | Describe medical variables, e.g. uncontrolled diabetes, osteoporosis, smoking, pharmacological treatment etc. If applicable, present the number of patients per subgroup.  Describe dental variables, e.g. periodontal disease, loss of teeth etc. If applicable, present the number of patients per subgroup. |
| Setting | Describe the setting of the research study, e.g. dental school. If applicable, present the number of patients per subgroup. |
| Co-existent conditions | Describe possible past interventions or co-interventions, previous interventions etc. If applicable, present the number of patients per subgroup. |
| Previous testing | Describe possible previous testing procedures prior to conducting the diagnostic test that could have changed the spectrum of the patients.  If applicable, present the number of patients per subgroup. |
| Severity of the target condition | Describe the severity of the target condition. The numbers of root glancing versus root penetration could be skewed.  If applicable, present the number of patients per subgroup. |
| Intended use of the index test | Assess whether the index test was used for assessing the target condition or for stability assessments of OMIs or both. If applicable, present the number of patients per subgroup. |
| Other comorbid  conditions | Describe other comorbid conditions, e.g., change in patient’s health ? If applicable, present the number of patients per subgroup. |

*Locate where information on each item can be found, e.g., Page 12 column 3.

**QUADAS-2 Collection form 3. Domain 2: Index test-Implants “risk of bias” [56,72,82]***

| **Entry** | **Description and character of the information** |
| --- | --- |
| Number of implants | Present the total number of implants and if applicable the number of implants per subgroup. |
| Implant type | Present the type of implant (code number) with the name of the company(ies) and if applicable the number of implants per subgroup. |
| Implant form | Describe the form of the screw: tapered/cylindrical/or a combination of forms.  Describe these specifics for each part of the screw. |
| Implant material | Describe the chemical make-up of the screw. |
| Diameter (D)  Length (L) | Describe the thread diameter and core diameter in mm.  Describe the thread length and body length in mm. |
| Surface finish | Describe the type of surface finish. |
| Drilling design | Describe if the screw has a self-drilling or a pre-drilling design. |
| Thread characteristics | Describe the width and angles of the crest of the screw. |
| Pitch dimensions | Describe the dimensions of the pitch of the screw. |
| Flute in tip | Describe if the tip of the screw is fluted or not.  Describe the length of the flute. |
| Flute in core | Describe if the core of the screw is fluted or not.  Describe the length of the flute. |
| Sterilized | Describe if the screws are sterilized by the company or by the operator. |

*Locate where information on each item can be found, e.g., Page 12 column 3.

**QUADAS-2 Collection form 4. Domain 2: Index test-Location “risk of bias” [56,72,82]***

| **Entry** | **Description and character of the information** |
| --- | --- |
| Implant site | Describe the exact insertion site of the implant. |
| Distance to root | Describe the distance between the implant and the root of a tooth in mm. |
| Assessment of bone condition | Describe if any quality assessment of the bone e.g. radiographically was undertaken prior to surgery. |
| Bone thickness | Describe the thickness of the cortical and trabecular bone in mm. |
| Keratinized/non keratinized mucosa | Describe if the implant was placed in the keratinized or non keratinized mucosa. |
| Mucosal thickness | Describe the thickness of the mucosa in mm. |
| Exposed/non exposed | Describe if the implant was left exposed or non exposed under the mucosa after surgery. |

*Locate where information on each item can be found, e.g., Page 12 column 3.

**QUADAS-2 Collection form 5. Domain 2: Index test-Surgery “risk of bias” [56,72,82]***

| **Entry** | **Description and character of the information** |
| --- | --- |
| Experience of the operator | Describe the number of mini-implants inserted by the operator prior to this study. |
| One or multiple operators | Describe if all surgeries were conducted by the same operator and if not how many implants were placed by each operator. |
| Flap or flapless surgery | Describe if surgery was flap or flapless. |
| Self-drilling or pre-drilling insertion technique | Describe if surgery was conducted according to the self-drilling or pre-drilling insertion technique. |
| Pilot holes for the self-drilling technique | Describe if pilot holes were drilled for the self-drilling technique and describe diameter and depth in mm. |
| Pre-drilling device for the self-drilling pilot holes | Describe the type of device that was used for the self-drilling pilot holes. |
| Pre-drilling speed for the selfdrilling pilot holes | Describe the pre-drilling speed in rpm (rounds per minute) for the self-drilling pilot holes. |
| Pilot holes for the pre-drilling technique | Describe if pilot holes were drilled for the predrilling technique and describe the diameter and depth in mm. |
| Pre-drilling device for the pre-drilling pilot holes | Describe the type of device that was used for the pre-drilling pilot holes. |
| Pre-drilling speed for the predrilling pilot holes | Describe the pre-drilling speed in rpm (rounds per minute) for the predrilling pilot holes. |
| Distance between the screws | In the case of multiple screws, describe the distances between the screws in mm. |
| Insertion depth | Describe the insertion depth in mm. |
| Friction between the head of the screw and the bone | Describe if the screw head touched the bone and had caused friction. Such a contact could have caused stripping of the bone. |
| The direction of insertion | Describe the angulation of insertion of the screw in degrees. |
| Insertion speed | Describe the insertion speed in rpm (rounds per minute). |
| Axial insertion force | Describe the axial insertion force in kg. |
| Insertion with insertion torqueing device | Describe if the insertion was conducted with a torqueing device. |
| Type of torqueing device | Describe the type of the insertion torqueing device and describe if it was a mechanical or a digital device. |
| Stripping of the bone | Describe if stripping occurred (rotation of the screw without penetration of the bone). |
| Mono or bicortical anchorage | Describe if the anchorage was mono or bicortical. |

*Locate where information on each item can be found, e.g., Page 12 column 3.

**QUADAS-2 Collection form 6. Domain 2: Index test-collected items “risk of bias” and “concerns of applicability” [56,72,82]***

| **Entry** | **Description and character of the information** |
| --- | --- |
| Implant, location, or surgery related factors | Describe implant, location or surgery related factors that could have caused biased outcomes of the index test.  (extracted in data collection forms 6-8) |
| Experience of the operator | Describe the experience of the operator, i.e., the operator that inserts the implant with the digital torque sensor. |
| Foreknowledge of the results of the reference standard | Describe whether operators had foreknowledge of results of the reference standard.  This entry is never applicable for our index test, because this test is always conducted prior to the reference standard. |
| Foreknowledge of intermediate test results | Describe whether operators had foreknowledge of results of intermediate test results, intermediate recordings with the index test, i.e., insertion torque or other intermediate tests, e.g., resonance frequency assessments. |
| Pre-specified threshold of the index test | Describe what the threshold of the index test is and how and when it was established, i.e., a priori or post hoc. |
| Timing of outcome measurement | Describe when the outcome (maximum insertion torque) was recorded, i.e. maximum torque during the entire insertion process or maximum torque during the final rotation of the screw. |
| Quality torque recording | Describe the quality of the torque recording, e.g., conducted with a mechanical or a digital device. |
| Number of outcome assessors | Describe the number of outcome assessors. Ideally more than one operator assesses the outcomes in order to avoid inadequate readings or exclusions of readings. |
| Calibration of the torque sensor | Describe whether the torque sensor was calibrated and how this procedure was conducted. |
| Standardization of the index test | Describe whether always the same instrument was used for all recordings with the index test. |
| Conflict of interest | Describe whether the authors or any of the stakeholders had a conflict of interest for using a specific index test and whether this could have led to biased outcomes. |
| Other procedural variables | Describe whether other procedural variables could have influenced the outcomes of the index test, e.g., pain, high number of implant fractures, non-continuous implant insertion etc. |

*Locate where information on each item can be found, e.g., Page 12 column 3.

**QUADAS-2 Collection form 7. Domain 3: Reference standard “risk of bias” and “concerns of applicability” [56,72,82]***

| **Entry** | **Description and character of the information** |
| --- | --- |
| Experience of the operator | Describe the experience of the operator, i.e., the operator that records the reference standard, e.g., the radiology technician |
| Foreknowledge of the results of the index test | Describe whether operators had foreknowledge of results of the index test. |
| Foreknowledge of intermediate test results | Describe whether operators that conduct the reference standard had foreknowledge of results of intermediate test results, intermediate recordings with the index test, i.e., insertion torque or other intermediate tests, e.g., resonance frequency assessments. |
| Quality reference standard | Describe the quality of the reference standard, e.g., 2D versus 3D radiographs. Assess whether the quality of the reference standard is sufficient to provide an accurate reading. 3D images are the preferred standard |
| Number of outcome assessors | Describe the number of outcome assessors. Ideally more than one operator assesses the outcomes in order to avoid inadequate readings or exclusions of readings. |
| Calibration of the reference standard | Describe whether the reference standard was calibrated and how this procedure was conducted. |
| Calibration of the operators | Describe whether operators were calibrated prior to classifying the target condition and how this procedure was conducted |
| Intra-or inter-operator differences | Present the intra-or inter operator differences. Assess whether these differences are small enough to be ignored. |
| Standardization of the reference standard | Describe whether always the same instrument was used for all recordings with the reference standard. |
| Co-interventions | Describe whether co-interventions were conducted between the index test and the reference standard. Assess whether these interventions could influence the results of the reference standard. |
| Defining the target condition | Describe how the target condition was defined, e.g., no root contact, root contact without root penetration, root contact with penetration. |
| Conflict of interest | Describe whether the authors or any of the stakeholders had a conflict of interest for using a specific reference standard and whether this could have led to biased outcomes. |
| Other procedural variables | Describe whether other procedural variables could have influenced the outcomes of the reference standard. |

*Locate where information on each item can be found, e.g., Page 12 column 3.

**QUADAS-2 Collection form 8. Domain 4: Flow and timing “risk of bias” [56,72,82]***

| **Entry** | **Description and character of the information** |
| --- | --- |
| Time interval index test reference standard | Describe the time interval between the index test(s) and the reference standard. Describe whether this interval was correct or too long. |
| All patients both tests | Describe whether all patients received both the index test and the reference standard. Control in flow diagram. |
| Inclusion in analysis | Describe whether all patients tested were also included in the data analysis |
| Withdrawals | Describe whether patients withdrew and present the explanation for this withdrawal. |

*Locate where information on each item can be found, e.g., Page 12 column 3.
