# Supplementary material for: Insertion torque recordings for the diagnosis of contact between orthodontic mini-implants and dental roots: protocol for a systematic review
Source: Syst Rev. 2015 Apr 2;4:39. doi: 10.1186/s13643-015-0014-6 (PMC4407834; doi:10.1186/s13643-015-0014-6)
Supplement: Additional file 4: — QUADAS-2 quality assessment tool tailored to the clinical question [80,82]. The tailored and pilot tested QUADAS-2 tool. [file 13643_2015_14_MOESM4_ESM.docx]

**Additional file 4**

**QUADAS-2 Quality assessment tool tailored to the clinical question [80,82]**

**Phase 1: State the review question:***

| **Patients (setting, intended use of index test, presentation, prior testing):** |
| --- |
| **Index test(s):** |
| **Reference standard and target condition:** |

*The review question and the eligibility criteria are defined in the protocol of this systematic review

**Phase 2: Draw a flow diagram for the primary study**

|  |
| --- |

**Phase 3: Risk of bias and applicability judgments**

QUADAS-2 is structured so that 4 key domains are each rated in terms of the risk of bias and the concern regarding applicability to the research question (as defined above). Each key domain has a set of signaling questions to help reach the judgments regarding bias and applicability.

| **DOMAIN 1: PATIENT SELECTION** |
| --- |
| **A. Risk of Bias**  **Describe methods of patient selection:**   \|  \| \| --- \|   **Signaling questions regarding the patients selected in the research study:**   \| Was a consecutive or random sample of patients enrolled ? \| Yes/No/Unclear \| \| --- \| --- \| \| Was a case-control design avoided ? \| Yes/No/Unclear \| \| Were the total number of patients adequate to answer the research  question ? (Was a power calculation conducted ?) \| Yes/No/Unclear \| \| Did the study avoid inappropriate exclusions ? \| Yes/No/Unclear \|   **Could the selection of patients have introduced bias ? RISK: LOW/HIGH/UNCLEAR**  **B. Concerns regarding applicability**  **Describe included patients (previous testing, severity of the target condition, intended use of the index test, demographic features, presence of differential diagnosis or comorbid conditions, setting of the study):**   \|  \| \| --- \|   **Signaling questions regarding the patients selected in the research study compared to those eligible for the review questions :**  (These signaling questions are only used to facilitate making a judgment regarding applicability)   \| Could demographic features, gender, age, ethnicity, knowledge of languages, socio-economic factors, location, disabilities, and mobility up to the moment of starting treatment in the selected patients influence the applicability ? \| Yes/No/Unclear \| \| --- \| --- \| \| Could the medical and dental health conditions up to moment of conducting the index test, e.g., the medical and dental health status in the selected patients influence the applicability ? \| Yes/No/Unclear \| \| Could the setting of the selected patients influence applicability ? \| Yes/No/Unclear \| \| Could co-existent conditions in the selected patients influence applicability ? \| Yes/No/Unclear \| \| Could previous testing in the selected patients influence applicability ? \|  \| \| Could the severity of the target condition in the selected patients influence applicability ? \| Yes/No/Unclear \| \| Could the intended use of the index in the selected patients influence applicability ? \| Yes/No/Unclear \| \| Could other comorbid conditions up to the moment of conducting the index test in the selected patients influence applicability, e.g., change in patient’s health, implant (e.g., implant design and dimensions) ? \| Yes/No/Unclear \|     **Is there concern that the included patients CONCERN: LOW/HIGH/UNCLEAR**  **do not match those targeted by the review question ?** |

| **DOMAIN 2: INDEX TEST(S)** |
| --- |
| **If more than one index test was used, please complete for each test**  **A. Risk of Bias**  **Describe the index test and how it was conducted and interpreted:**   \|  \| \| --- \|   **Signaling questions regarding the selected index test in the research study:**   \| Were biased outcomes of the index test caused by implant, location or surgery related factors avoided ? \| Yes/No/Unclear \| \| --- \| --- \| \| Was the insertion procedure (which includes the torque recording with the index test) conducted by an experienced operator(s) ? \| Yes/No/Unclear \| \| Were the index test results interpreted without knowledge of the results of the reference standard ? \| Yes/No/Unclear \| \| Were the index test results interpreted without knowledge of other intermediate test results ? \| Yes/No/Unclear \| \| If a threshold was used, was it pre-specified ? \| Yes/No/Unclear \| \| In the research study is the outcome, insertion torque, measured during the final rotation of the screw ? \| Yes/No/Unclear \| \| Is the quality of the torque recording accurate (assess the quality of the torque sensor, e.g., mechanical versus digital, design, easy to use) ? \| Yes/No/Unclear \| \| Was the outcome of the index test assessed by more than one outcome assessors ? \| Yes/No/Unclear \| \| Was the torque sensor calibrated ? \| Yes/No/Unclear \| \| Were always the same instruments used for the index test ? \| Yes/No/Unclear \| \| Was there no conflict of interest in using the index test ? \| Yes/No/Unclear \| \| Were there no other procedural variables that could have influenced the outcomes of the index test, e.g., implant, location, and surgery related factors. \| Yes/No/Unclear \|   **Could the conduct or interpretation of the index test RISK: LOW/HIGH/UNCLEAR**  **have introduced bias ?**  **B. Concerns regarding applicability**  **Describe variations in test technology, execution, or interpretation of a test:**   \|  \| \| --- \|   **Is there concern that the index test, its conduct, CONCERN: LOW/HIGH/UNCLEAR**  **or its interpretation differ from the review question ?** |

| **DOMAIN 3: REFERENCE STANDARD** |
| --- |
| **A. Risk of Bias**  **Describe the reference standard and how it was conducted and interpreted:**   \|  \| \| --- \|   **Signaling questions regarding the selected reference standard in the research study:**   \| Was the reference standard conducted by an experienced operator(s) ? \| Yes/No/Unclear \| \| --- \| --- \| \| Were the reference standard results interpreted without knowledge of the results of the index test(s) ? \| Yes/No/Unclear \| \| Were the reference standard results interpreted without knowledge of other intermediate test results ? \| Yes/No/Unclear \| \| Is the reference standard likely to correctly classify the target condition (compare 2D versus 3D radiographs)? \| Yes/No/Unclear \| \| Did more than 1 operator classify the target condition ? \| Yes/No/Unclear \| \| Was the reference standard machinery calibrated ? \| Yes/No/Unclear \| \| Were the operators calibrated prior to classifying the target condition ? \| Yes/No/Unclear \| \| Were intra-or inter-operator differences in classifying the target condition sufficiently small that they can be ignored ? \| Yes/No/Unclear \| \| Were always the same instruments used for the reference standard ? \| Yes/No/Unclear \| \| Were no specific interventions conducted between the index test(s) and the reference standard that could influence the diagnosis of the target condition ? \| Yes/No/Unclear \| \| Did the study present a clear definition of the target condition ? \| Yes/No/Unclear \| \| Was there no conflict of interest in using the reference standard ? \| Yes/No/Unclear \| \| Were there no other procedural variables that could have influenced the outcomes of the reference standard ? \| Yes/No/Unclear \|   **Could the reference standard, its conduct, or its RISK: LOW/HIGH/UNCLEAR**  **interpretation have introduced bias ?**  **B. Concerns regarding applicability**  **Describe the target condition**   \|  \| \| --- \|   **Is there concern that the target condition CONCERN: LOW/HIGH/UNCLEAR**  **as defined by the reference standard does not**  **match the review question ?** |

| **DOMAIN 4: FLOW AND TIMING** |
| --- |
| **A. Risk of Bias**  **Describe any patients who did not receive the index test(s) and/or reference standard or who were excluded from the 2 x 2 table (refer to flow diagram):**   \|  \| \| --- \|   **Describe the time interval and any interventions between index test(s) and reference standard:**   \|  \| \| --- \|   **Signaling questions:**   \| Was there an appropriate interval between index test(s) and reference standard ? \| Yes/No/Unclear \| \| --- \| --- \| \| Did all patients (implants) receive a reference standard? \| Yes/No/Unclear \| \| Did all patients (implants) receive the same reference standard ? \| Yes/No/Unclear \| \| Were all patients (implants) included in the analysis ? \| Yes/No/Unclear \| \| Were withdrawals explained ? \| Yes/No/Unclear \|   **Could the patient flow have introduced bias ? RISK: LOW/HIGH/UNCLEAR** |
