# Supplementary material for: Insertion torque recordings for the diagnosis of contact between orthodontic mini-implants and dental roots: protocol for a systematic review
Source: Syst Rev. 2015 Apr 2;4:39. doi: 10.1186/s13643-015-0014-6 (PMC4407834; doi:10.1186/s13643-015-0014-6)
Supplement: Additional file 5: — Tabular presentation for QUADAS-2 results of the selected studies [80]. Exemplary table for the presentation of the QUADAS-2 scores. [file 13643_2015_14_MOESM5_ESM.doc]

**Additional file 5. Tabular presentation for QUADAS-2 results of the selected studies (QUADAS-2 2014) [80]**

| **STUDY** | **RISK OF BIAS** | | | | **APPLICABILITY CONCERNS** | | |
| --- | --- | --- | --- | --- | --- | --- | --- |
|  | **Patient selection** | **Index test** | **Reference standard** | **Flow and timing** | **Patient selection** | **Index test** | **Reference standard** |
|  |  |  |  |  |  |  |  |
|  |  |  |  |  |  |  |  |

☺ Low ☹ High ? Unclear
