# Supplementary material for: Insertion torque recordings for the diagnosis of contact between orthodontic mini-implants and dental roots: protocol for a systematic review
Source: Syst Rev. 2015 Apr 2;4:39. doi: 10.1186/s13643-015-0014-6 (PMC4407834; doi:10.1186/s13643-015-0014-6)
Supplement: Additional file 6: — Protocols for conducting meta-analyses and assessing statistical heterogeneity. Protocol for conducting a meta-analysis for the primary and secondary research questions and models for investigating statistical heterogeneity. [file 13643_2015_14_MOESM6_ESM.docx]

**Additional file 6.**

**Protocols for conducting meta-analyses and assessing statistical heterogeneity**

**Protocol for conducting meta-analyses**

In Cochrane diagnostic accuracy reviews 2 strategies for summary statistics are used: (1) estimating a summary sensitivity and specificity at a chosen common threshold . Studies that do not report these values at any of these thresholds will be excluded from the meta-analysis [88]; (2) estimating the expected summary Receiver Operating Characteristic (ROC) curve across a variety of thresholds. This analysis only includes one threshold per study [88].

The choice between these 2 strategies will depend on the variation of these thresholds in the eligible studies [88]. If both strategies could complement each other, SROC curves and average operating points will be both displayed. Revman 5.3 will be used to create these figures for each experimental model and for each specific analysis [89]. The summary ROC plots will present a single sensitivity-specificity point with its confidence intervals for the outcomes of each individual study [88]. When pooling of outcomes is possible, summary statistics will be added to these graphs. Paired forest plots will present the sensitivity and specificity together with their 95% confidence intervals [88]. In these plots, studies will be ordered to facilitate a visual representation of a possible association between sensitivity and specificity. If such an association is lacking, studies will be presented in alphabetical order [88]. Summary statistics will not be added to these coupled forest plots [88].

A random effects analysis will be the most suitable model for the meta-analysis, because heterogeneity is expected in studies of diagnostic test accuracy. The fixed effect model will only be used when the variability between studies is small and explicable by chance [88]. Review manager 5.3 will be used for the meta-analysis, but a statistician will also be consulted for potential external software [89].

For the secondary research question, we will conduct a fixed-effect meta-analysis when the effects of interventions of all eligible studies are similar in both magnitude and direction. Random effects models are applied when the effect sizes are not identical, but related and follow some distribution [90]. The mean difference between maximum insertion torque values with and without root contact will be the effect measure for this meta-analysis. Review manager 5.3 will also be used for the meta-analysis for the secondary research question [89].

**Protocol for assessing statistical heterogeneity**

For the primary research question both the bivariate and hierarchical SROC (HSROC) models will be used to investigate heterogeneity by assessing the effects of covariates on summary sensitivity and specificity and the shape and position of the summary ROC curve [88,92,93]. When only a small number of studies will be available these statistical models will be either avoided or used with caution [88].

For the secondary research question a variety of statistics: Q, p, T^2^, T, and I^2^ are used to test for heterogeneity [90,94-97]. In the presence of minimal, moderate, or substantial heterogeneity, i.e., I^2^ in the range of 0-90%, the dispersion of outcomes will be presented in a forest plot with or without the summary diamond [90,91,98]. The following strategies will be considered for dealing with (statistical) heterogeneity [90]: 1) controlling the correctness of the extracted data; 2) not conducting a meta-analysis. In the presence of an I^2^ higher than 90%, a narrative format will be applied for this systematic review, because conducting a meta-analysis in the presence of excessive heterogeneity could mislead the reader [91,99]; 3) further exploring the causes of heterogeneity and its impact on the treatment effects through subgroup analyses and meta-regression [90]; 4) ignoring heterogeneity by applying a fixed-effect meta-analysis; 5) incorporating he+terogeneity by applying a random-effects meta-analysis; 6) changing the effect measure, e.g., recording torque values at different time points during the insertion process; 7) excluding studies [90].
